# Cardiovascular Disease in Ageing: An Overview on Thoracic Aortic Aneurysm as an Emerging Inflammatory Disease

**DOI:** 10.1155/2017/1274034

**Published:** 2017-10-24

**Authors:** Calogera Pisano, Carmela Rita Balistreri, Alessandro Ricasoli, Giovanni Ruvolo

**Affiliations:** ^1^Cardiac Surgery Unit, “P. Giaccone” University Hospital, Palermo, Italy; ^2^Department of Pathobiology and Medical and Forensic Biotechnologies, University of Palermo, Palermo, Italy; ^3^Cardiac Surgery Unit, Tor Vergata University Hospital, Rome, Italy

## Abstract

Medial degeneration associated with thoracic aortic aneurysm and acute aortic dissection was originally described by Erdheim as a noninflammatory lesion related to the loss of smooth muscle cells and elastic fibre fragmentation in the media. Recent evidences propose the strong role of a chronic immune/inflammatory process in aneurysm evocation and progression. The coexistence of inflammatory cells with markers of apoptotic vascular cell death in the media of ascending aorta with aneurysms and type A dissections raises the possibility that activated T cells and macrophages may contribute to the elimination of smooth muscle cells and degradation of the matrix. On the other hand, several inflammatory pathways (including TGF-*β*, TLR-4 interferon-*γ*, chemokines, and interferon-*γ*) seem to be involved in the medial degeneration related to aged and dilated aorta. This is an overview on thoracic aortic aneurysm as an emerging inflammatory disease.

## 1. Introduction

**Figure 1:**
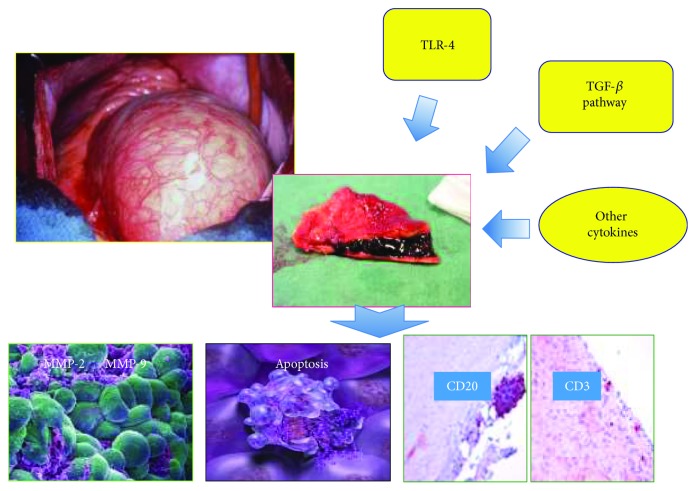
Thoracic aortic aneurysm as an emerging inflammatory disease.

The average lifespan of the human population is increasing worldwide, mostly because of declining fertility and increasing longevity. It has been predicted that, in 2035, nearly one in four individuals will be 65 years or older [1]. As supported by the growing evidence, age constitutes the crucial risk factor for the development of cardiovascular diseases (CVDs). Accordingly, their prevalence dramatically increases with increasing age [2]. In such association, emerging data that are underlining the crucial involvement of pathophysiological pathways in cardiovascular ageing, dysfunction, and CVD onset are also confirmed by literature [1–6]. Insights and advances in this field are encouraged. This might permit of identifying pathways closely implicated in cardiovascular pathophysiology and useful for improving management, outcomes, and prevention of human CVDs. Today, it is possible to distinguish diverse levels of ageing in cardiovascular system. For instance, in 2017, Steenman and Lande summarized four levels of ageing of heart: functional, structural, cellular, and molecular [7]. Functional changes in the aged heart include systolic and diastolic dysfunction and alterations of the cardiac electrical system [8–11]. Structural changes determine an increase in thickness of heart [12, 13] and vessel's wall [14]. Cellular changes include fibrosis [15], medial necrosis and apoptosis [16], and amyloid deposition [17]. Furthermore, the involvement of many molecular pathways implicated in cardiac aging is emerging, even if their role is well-discovered and understood. In regard to vascular system, the main vessel, that is the aorta, as the entire cardiovascular system or any other organ, tissue, and system of human body, shows several modifications with advancing age [18] in all its sections. In particular, in thoracic aorta section, object of our dissertation, diverse age-related changes occur and are responsible for onset of thoracic aorta dysfunction and pathological entities, including medial degeneration (MD) and remodelling. These last assume the role of the catalytic and accelerator drivers in the onset and progression of pathological complications, including the first and more common, the sporadic thoracic ascending aneurysm (S-TAA) [19]. This leads to evidence that S-TAA risk increases when one reaches 65 years old. However, numerous gaps related to the effective cellular and molecular mechanisms remain to be resolved. In this context, the detection of specific histopathological MD phenotypes in old people, associated with a major risk for S-TAA onset and its complications, might be of particular help. We have recently identified a particular MD phenotype significantly associated with the onset of S-TAA in aged individuals, even if based on a small sample size that we have suggested as a potential biomarker. Emerging literature data are also evidencing the key involvement of an upregulation of metalloproteinases (MMPs) and apoptosis, sterile chronic inflammation, and genetic factors in the complex pathophysiology of S-TAA [19]. They are in agreement with recent data, proposing that the sterile inflammation and the related mediators, including cytokines, MMPs, and death mediator, are the shared pathological mechanism for the onset of type A aortic dissection (TAD) [19, 20]. Based on these observations, we reported an overview on emerging literature data about the implication of inflammation in S-TAA pathophysiology Figure 1.

## 2. Inflammatory Features of Thoracic Ascending Aneurysm

Well-recognized variations in the onset and progression characterize TAA and abdominal aortic aneurysm (AAA). However, inflammation is emerging as a common mechanism in the complex, yet different, pathophysiology of the two types of aorta aneurysms [19]. Likewise to AAA, histological studies demonstrate the presence of high levels of inflammatory cells in the adventitia and media of thoracic aortic wall. In particular, it has been demonstrated that macrophages and T lymphocytes are prevalently observed in the thoracic aorta samples of patients affected by sporadic TAA [20–23]. Furthermore, a number of other studies documented the evidences of an increased apoptosis of smooth muscle cells (SMCs) in MD [24, 25]. Accordingly, immunohistochemical studies demonstrated that MD is associated with levels of p53, a Bax upregulation, and apoptosis of SMC in the aorta of patients with TAD [26]. High levels of Fas (cell surface death receptor) have been diffusely assessed into the aortic media and adventitia. T lymphocytes seem to be the cells responsible for FasL production, in contrast, Fas was found diffusely throughout the media and adventitia. These results suggest apoptosis of primarily SMCs through the Fas-FasL pathway in aneurysmal tissue [22]. The T lymphocyte and macrophage infiltration and TUNEL staining were more pronounced in the media of the dissected aortas than in the aneurysmal aortas. The dissection of blood into the wall of the aorta is a traumatic tissue injury, and it is, therefore, not surprising that inflammatory and apoptotic markers would be increased in the damaged tissue [22]. Although both inflammatory and TUNEL-positive cells were observed along the margin of the dissections, the inflammatory cells and apoptotic markers were present throughout the media as well. It is possible that the degree of inflammation and apoptosis in the media may be increased in an aorta before dissection. These events could potentially lead to increase the rates of loss of SMCs and the destruction of the tissue, setting the stage for the dissection to occur, despite the aortic dimensions. Accordingly, unlike the current recommendations [27], recent studies demonstrated that the diameter is not the only parameter that needs to be considered in order to prevent dissection [24, 25, 28]. Some individuals have dissection when the aorta is minimally enlarged, and it is necessary to determine whether this premature dissection is related to the degree of inflammation and apoptosis in the aortic media. In fact, it seems that patients who developed premature dissection carried a particular genetic risk profile characterized by single-nucleotide polymorphisms (SNPs) in inflammatory genes (-786T/C eNOs, D/IACE, -1562 C/T MMP-9, and -735C/T MMP-2) that causes an important inflammatory reaction, severe tissue injury, and vascular matrix remodelling [29–33].

## 3. Role of Cytokines in TAA Pathogenesis

Several cytokines seem to be involved in TAA formation and complications (rupture and dissection). Among these, transforming growth factor beta (TGF-*β*) is recognized to have a crucial role in S-TAA pathogenesis, causing extracellular matrix (ECM) degeneration through the production of plasminogen activators and the release of metalloproteinases-2 (MMP-2) and metalloproteinases-9 (MMP-9) [34]. TGF-*β* family consists of TGF-*β*1, TGF-*β*2, and TGF-*β*3 members, which are pleiotropic-secreted cytokines having a broad spectrum of biologic functions. Among these, the TGF-*β*1 has numerous cellular functions, including cell growth, cell proliferation, cell differentiation, and apoptosis. In humans, TGF-*β*1 can stimulate or inhibit cell growth in diverse cellular and tissue targets. In addition, TGF-*β*1 can modulate cell differentiation and proliferation through both canonical and noncanonical pathways [35]. The canonical pathway involves the activation of the TGF-*β* receptor 1, the phosphorylation of regulatory small mother against decapentaplegic proteins (SMADs 2 and 3), the recruitment of SMAD4 to form a SMAD2/3-SMAD4 complex, the importation into the nucleolus, and activation of gene transcription [36]. Normally, there is an autoregulatory feedback loop on TGF-*β* signalling provided by the canonical pathway [37]. However, it is believed that the loss of functional mutations in genes encoding TGF-*β* pathway leads to an upregulation of TGF-*β* signalling, especially through the noncanonical pathways due to loss of this feedback inhibition. The resulting upregulation in TGF-*β* is considered to be a crucial condition strongly associated with the formation of TAA. Based on these observations, we recently assessed the role of five genetic variants of TGF-*β* pathways (TGF-*β*1 and 2 isoforms and R1 and R2 receptors) in sporadic TAA. The most relevant finding of this study has allowed us to propose that rs900 TGF-*β*2 SNP is associated with sporadic TAA in women [34]. On the other hand, other reports [38–40] have evidenced a direct or an indirect central role to TGF-*β*2 and its genetic variants in the pathogenesis of both syndromic and familial TAAs. In addition, it has been shown that mutations in the TGFBRII genes deregulate the TGF-*β*2 signalling pathway involved in TAA pathogenesis. TGF-*β*2 gene mutations have been found in familial TAAs and dissections associated with mild systemic features of Marfan syndrome and Loeys-Dietz syndrome and in TAA and dissection associated with mitral valve disease [41]. However, the exact role of TGF-*β*2 in TAA pathogenesis is not clear. In particular, both the genetically determined loss of function and the “paradoxical” augment in the downstream TGF-*β* signalling pathway might be important for TAA development [42]. Furthermore, vascular remodelling, characterizing thoracic aneurysm, seems prevalently to be the result not only of TGF-*β* pathways but also of upregulation of multiple cytokines, including interleukin-10 (IL-10) [43], an anti-inflammatory cytokine able to modulate the activity of TGF-*β* pathway, interleukin-6 (IL-6), interleukin-8 (IL-8), tumour necrosis factor- (TNF-) *α*, and monocyte chemoattractant protein-1 [44–46]. On the other hand, interesting studies revealed that circulating levels of interferon-*γ*, interferon-*γ*-induced chemokines, interferon-inducible T-cell alpha chemoattractant, interferon-inducible protein-10, and monokine induced by interferon gamma are all increased in patients affected by TAAs, even if the levels of these chemokines do not correlate with the size of the aneurysms [47]. Conversely, IL-4 levels have been reported to be reduced in TAA patients [48].

## 4. Other Inflammatory Biomarkers

Systemic levels of inflammatory mediators have been assessed in both acute aortic syndromes (AAD) and TAAs. C-reactive protein (CRP) levels have been observed to increase during AADs [49]. However, it is not clear whether the systemic CRP level increase starts before the onset of these complications or if it is an effect. Regarding this aspect, some studies have shown that a substantial portion of patients with TAA shows normal CRP levels on hospital admission [50]. In contrast, whereas the recent evidence suggests that CRP is increased even in the very early hours after the onset of symptoms [45], this, together with the difficulty in differentiating acute aortic syndromes from other acute cardiovascular problems, such as pulmonary embolism and myocardial infarction, has limited the clinical application of this biomarker for diagnostic purposes. However, the presence of an inflammatory state in both acute aortic syndromes and, to a lesser degree, in chronic TAAs and chronic ADs appears clear. This is evidenced by the increases in both CRP and white blood cell counts [51]. In addition, CRP can be useful for predicting long-term results of acute aortic syndromes. Accordingly, in fact, previous studies have shown that growing CRP levels on admission are associated with a poor prognosis in this setting and that this marker strongly correlates with adverse long-term events at follow-up in Chinese people in both type A (together with white blood cell count) [52] and type B AADs; in this latter case, the most powerful predictor is the CRP peak level after the acute event [53]. Another crucial biomarker is the plasma D-dimer (DD), which is a product of plasmin fibrinolysis of cross-linked fibrin, representing an indirect demonstration of coagulation activation leading to increased fibrin turnover. DD is commonly used for the diagnosis or the exclusion of deep venous thrombosis and pulmonary embolism. However, it is the most studied and likely the most reliable biochemical diagnostic tool in AADs. As an AAD biomarker, DD is quite nonspecific since it can rise in a number of disease states, such as malignancies, recent surgery, disseminated intravascular coagulation, deep venous thrombosis, or recent trauma. Nevertheless, several prospective and retrospective studies [54, 55] have shown that, with a threshold value of 0.4–0.5 *μ*g/ml, AADs can be diagnosed with a high sensitivity, approximately 94–100%, but with a much lower specificity (35–75%). Interestingly, data from the IRAD-Bio registry suggest that DD has good sensitivity, even in the case of early (<6 h) presentation [56], although some evidences suggest that testing patients with this biomarker may result in missing the diagnosis of AAD in up to 18% of patients [57]. Another major issue about DD is that the assessment of this marker can give a negative result either in the case of a thrombosed false lumen or in the case of an intramural haematoma [58–60]. This further feature limits the chances of using DD, being a solid watershed test for acute aortic syndromes. However, DD may become a more powerful biomarker for monitoring false lumen evolution over time, as hypothesized by some studies [61, 62]. These last evidenced a possible relationship between DD levels and presence of blood flow in the false lumen. Furthermore, a recent meta-analysis [63] of observational studies has suggested some new point on the role of DD in the diagnosis of AD, showing that the best DD performing the cut-off level is 0.5 *μ*g/ml, with a sensitivity of 97% and a negative predictive value of 96%. On the basis of the results of this meta-analysis, a level of 0.5 *μ*g/ml might be proposed as a rapid and useful screening tool to exclude AADs. This could be of help for the physician in deciding whether to order additional imaging studies to confirm or refuse the diagnosis of AD.

## 5. Tole of Toll-Like Receptor-4 in Thoracic Aneurysm Pathogenesis

Recent evidences underlie the important role of the Toll-like receptor-4 (TLR-4) as key inflammatory promoter for sporadic TAA [64, 65]. It is a member of Toll-like receptor (TLRs) family. TLRs are the most extensively studied innate receptors, and their roles in innate and adaptive immunity are well-documented [66, 67]. The TLR-4 originally was described as part of the first-line defence against Gram-negative bacteria, it is expressed on leukocytes and a large array of tissue and cell types, such as aortic wall cells, particularly endothelial cells (ECs) and vascular smooth muscle cells (VSMCs), and it responds to particular damage-related products activating a particular inflammatory reaction. In the specific case of sporadic TAA, TLR-4 evocates and modulates the expression and activation of endothelium dysfunction and remodelling aorta pathways. In particular, we suggested a model [68], in which the overstate activation of TLR-4 induced by high levels of danger-associated molecular patterns (DAMPs) determines a typical phenotypic switching of EC and VSMC cells, with the involvement of other pathways, such as stress and stretch pathways. This implies the activation of nuclear factor-*κ*B (NF-*κ*B) transcription factor and the production and release of the so-called arterial-associated senescence secretor phenotype (AASSP) characterized by numerous inflammatory mediators, mitotic and trophic factors, proteoglycans and MMPs such as MMP-2 and MMP-9, and vasoactive molecules [31]. In addition, this induces the reduction of nitric oxide (NO) as well. This complex scenario results in modifications of vascular tone and permeability and degradation of components of extracellular matrix (ECM) and elastic fragmentation. Vascular remodelling and MD are, hence, evocated, which can evolve in aneurysm, dissection, and rupture of the aorta wall.

## 6. Evidences about the Role of Inflammation in TAA Complications

There are several experimental evidences that support inflammatory cells and cytokines as the cause of TAA complications. In a recent study, Tieu et al. [69] showed that subcutaneous infusion of angiotensin II, a vasopressor known to promote vascular inflammation, induced aortic production of the proinflammatory cytokine IL-6 and the monocyte chemoattractant MCP-1 into older C57BL/6J mice. The production of these factors occurred predominantly in the tunica adventitia, along with macrophage recruitment, adventitial expansion, and development of thoracic and suprarenal aortic dissections. In contrast, a reduced dissection incidence was observed after angiotensin II infusion into mice lacking either IL-6 or the MCP-1 receptor CCR2. Further analysis revealed that angiotensin II induced CCR2 + CD14hiCD11bhiF4/80– macrophage accumulation selectively in aortic dissections and not in aortas from Il6−/− mice. Adoptive transfer of Ccr2+/+ monocytes into Ccr2−/− mice resulted in selective monocyte uptake into the ascending and suprarenal aorta in regions of enhanced ROS stress, with restoration of IL-6 secretion and increased incidence of dissection. In vitro, culture of monocytes and aortic adventitial fibroblasts produced MCP-1– and IL-6–enriched conditioned medium that promoted differentiation of monocytes into macrophages, induced CD14 and CD11b upregulation, and induced MCP-1 and MMP-9 expression. These results suggest that leukocyte-fibroblast interactions in the aortic adventitia potentiate IL-6 production, inducing local monocyte recruitment and activation, thereby promoting MCP-1 secretion, vascular inflammation, ECM remodelling, and aortic destabilization. On the other hand, Kurobe et al. [70] demonstrated that combined treatment with angiotensin II and *β*-aminopropionitrile induces degenerative AAs in wild-type mice and azelnidipine prevents aneurysm progression via its anti-inflammatory effect. The anti-inflammatory effect of azelnidipine caused the decreased expression levels of TNF-*α*, MMP-2, and MMP-9, and it conversely enhanced gene expression level of vascular SIRT-1. Vascular SIRT-1 possesses cardiovascular protective properties and improves the function of vascular smooth muscle and vascular endothelial cells [71]. Other experiments have been performed in rat model to investigate the inflammatory basis of TAA. Johnston et al. [72] induced TAA using elastace in mice deficient of IL-1*β* (IL-1*β* knockout) or IL-1 receptor (IL-1R knockout), and they concluded that genetic and pharmacological inhibition of IL-1*β* decreased TAA formation and progression, indicating that IL-1*β* may have an important role in TAA formation and complications. Accordingly, decreased IL-1*β* secretion by thoracic aortic cells in vitro with z-VAD suggests a critical upstream role for caspase in TAA IL-1*β* production. Z-VAD is a potent inhibitor of caspase, which cleaves inactive pro–IL-1*β* to its active form [73]. Caspase activation occurs as part of the inflammasome, a large multiprotein complex containing the NLRP3 protein responsible for caspase activation [74]. The NLRP3 inflammasome is known to play a role in multiple inflammatory conditions and is increased in TAAs [75]. The presence of NLRP3 and caspase in experimental TAA sections, along with the decrease in IL-1*β* production with caspase inhibition, suggests that caspase and the NLRP3 inflammasome are potential alternative target upstream of IL-1*β* production that may be useful for further investigation of TAA treatment in order to prevent deadly complications. Finally, another proof that the inflammation has an important role in TAA complications has been drawn by studies regarding mesenchymal stem cells (MSCs) for treatment of aortic aneurysm in rat [76]. The mechanism by which MSCs stabilized aneurysm and prevent dissection is related to their capacity to differentiate, in vitro, in vascular smooth muscle cells (VSMCs) with subsequent reduction of inflammatory reaction. In fact, the most accepted recent pathophysiological hypothesis on TAA proposes the key role of stress- and stretch-induced pathways both in endothelial cells (ECs) lining vasa vasorum and vascular aortic smooth muscle cells (VSMCs) activated by biochemical/mechanical inciting stimuli (i.e., first of all, hypertension, hemodynamic force, smoking and an overload of lipids, etc.) [77]. Their activation created a typical phenotypic switching both in EC and VSMC determining production and release of numerous mitotic and trophic factors, proteoglycans, and metalloproteinases (MMPs), such as MMP-2 and MMP-9 [31]. As result, different cellular and extracellular mechanisms are induced and determine sporadic TAA onset and progression as a multifactorial process with several steps. Among these, inflammatory/immune cellular infiltration in adventitia and media of the aortic wall, oxidative stress, and apoptosis appear principally involved [78]. Persistence of stress or stretch abnormalities results in continued upregulation of these pathways, and ultimately it leads to a proteoglycan accumulation, an increased vascular volume in vasa vasorum, and degradation of elastic fibres with consequent alterations of structure and composition of vascular ECM. Consequent manifestations are the dissection followed or associated with expansion and rupture of the aortic wall.

## 7. Conclusions

Thoracic aortic aneurysm might be advocated as a new emerging inflammatory disease. Evidences suggest that T lymphocytes and macrophages are the most important inflammatory cells involved in TAA pathogenesis. In addition, a lot of cytokines seem to be involved in the medial degenerative process (loss of smooth muscle cells, elastic fragmentation, and medionecrosis) that induces TAA progression and AAD onset. In particular, the TGF-*β* signalling pathways have been proposed as an important target involved in both syndromic and sporadic TAA. It is evident that a new therapeutic treatment with statin and anti-inflammatory drug should be proposed in order to prevent aneurysm progression and its lethal complication.
